# Comment on: Extracorporeal hemoadsorption in critically ill COVID‑19 patients on VV ECMO: the CytoSorb therapy in COVID‑19 (CTC) registry

**DOI:** 10.1186/s13054-023-04578-4

**Published:** 2023-07-24

**Authors:** A. Supady, D. L. Staudacher, T. Wengenmayer

**Affiliations:** 1 Interdisciplinary Medical Intensive Care, Medical Center, University of Freiburg, Hugstetter Straße 55, 79106 Freiburg, Germany; 2 Faculty of Medicine, University of Freiburg, Freiburg, Germany; 3 Heidelberg Institute of Global Health, University of Heidelberg, Heidelberg, Germany

Dear Editor,

With great interest, we read the report from the CytoSorb therapy in COVID‑19 (CTC) multicenter registry describing the use of extracorporeal hemoadsorption with the CytoSorb device in patients with severe COVID-19 supported with veno-venous extracorporeal membrane oxygenation (VV ECMO) [1]. We congratulate the authors for collecting and presenting interesting findings from this large multicenter cohort.

In the following, we would like to address some concerns with respect to the data presented and the interpretation of the results.

The authors describe the use of hemoadsorption with the CytoSorb adsorber to be associated with favorable survival rates and they suggest early initiation of CytoSorb treatment to be superior to delayed initiation. However, these strong claims are not supported by the data. Interpreting results from a comparison of retrospectively defined groups in an observational registry requires careful consideration, as results may be confounded by selection bias.

In this cohort, defining groups based on timing of initiation of CytoSorb is arbitrary. The “decision to use hemoadsorption therapy was at the discretion of the treating physicians”, therefore, systemic bias may have influenced the timing for initiation of CytoSorb. Patients in the late initiation group were older and age is a known risk factor for mortality in many ICU patients. Interestingly, patients in this group had less comorbidities, and PEEP, peak respiratory pressure and driving pressure were lower at baseline. These imbalances make it difficult to draw meaningful conclusions with regard to the impact of each of these factors on outcome parameters. Most importantly, in both groups, CytoSorb was started with or early after initiation of ECMO, therefore, the perceived between group differences could also be caused by differences in ECMO timing as previous evidence suggests that early initiation of ECMO might be superior to late initiation. Consequently, all findings from this registry study should primarily be used to generate hypotheses for future studies and not be used prematurely to inform treatment decisions and standards. Without a control group not receiving CytoSorb therapy, it is not possible to draw reliable conclusions about any additional effect or benefit of CytoSorb therapy.

Furthermore, these claims are in stark contrast to results from recent randomized-controlled trials and a recent review and meta-analysis. In these studies, no significant benefit of the use of CytoSorb on patient-centered outcomes has been identified. Neither did it reduce mortality nor did it significantly reduce the levels of inflammatory parameters [2–4].

The study describes the additional use of CytoSorb in COVID-19 patients on VV ECMO. However, only very limited treatment data after initiation of ECMO and CytoSorb are presented in this work. The authors explain the expected benefit of CytoSorb by supporting a strategy of “enhanced lung rest” to prevent development of ventilator-induced lung injury (VILI). Yet, besides baseline characteristics including p/F-ratio, PEEP, driving pressure and peak pressure no further data on ventilation strategies, ventilator or ECMO settings or blood gas parameters after initiation of ECMO and CytoSorb are presented. The extent to which the proclaimed strategy of lung rest ventilation was pursued in these patients and its impact on the outcome of the patients, can only be speculated.

ECMO is a highly invasive treatment option for patients with very severe respiratory failure. In previous observations, in a significant number of patients bleeding complications have been observed [5]. With respect to safety considerations, it would be interesting to learn more about bleeding and thromboembolic complications, including the requirement for ECMO circuit or component exchanges in this cohort in order to more comprehensively assess the safety profile of the intervention.

The authors correctly point out that the CytoSorb adsorber could also remove useful substances and drugs from the blood of the patients. Unfortunately, they miss to present analyses of blood concentrations of potentially adsorbed blood components or drugs before and after initiation of CytoSorb to be able to support the proclaimed safety or effectiveness.

Finally, the application of CytoSorb is not well described within this study. Relevant treatment details are missing, including the duration of CytoSorb, the number of adsorbers used for each patient and actual flow rates that have been achieved.

In conclusion, the interesting results presented from the CTC registry lack relevant information to support the conclusion of a favorable treatment effect of hemoadsorption in COVID-19 patients on ECMO and should therefore be interpreted very cautiously. The current body of evidence does not support the uncritical use of CytoSorb outside controlled clinical trials.

## Data Availability

Not applicable.

## References

[1] Hayanga JWA, Song T, Durham L, Garrison L, Smith D, Molnar Z, Scheier J, Deliargyris EN, Moazami N (2023). Extracorporeal hemoadsorption in critically ill COVID-19 patients on VV ECMO: the CytoSorb therapy in COVID-19 (CTC) registry. Crit Care.

[2] Becker S, Lang H, Vollmer Barbosa C, Tian Z, Melk A, Schmidt BMW (2023). Efficacy of CytoSorb®: a systematic review and meta-analysis. Crit Care.

[3] Monard C, Bianchi N, Poli E, Altarelli M, Debonneville A, Oddo M, Liaudet L, Schneider A (2023). Cytokine hemoadsorption with CytoSorb(®) in post-cardiac arrest syndrome, a pilot randomized controlled trial. Crit Care.

[4] Supady A, Weber E, Rieder M, Lother A, Niklaus T, Zahn T, Frech F, Muller S, Kuhl M, Benk C (2021). Cytokine adsorption in patients with severe COVID-19 pneumonia requiring extracorporeal membrane oxygenation (CYCOV): a single centre, open-label, randomised, controlled trial. Lancet Respir Med.

[5] Schmidt M, Langouet E, Hajage D, James SA, Chommeloux J, Brechot N, Barhoum P, Lefevre L, Troger A, de Chambrun MP (2021). Evolving outcomes of extracorporeal membrane oxygenation support for severe COVID-19 ARDS in Sorbonne hospitals, Paris. Crit Care.

